# Transmission of influenza A virus and porcine reproductive and respiratory syndrome virus using a novel nurse sow model: a proof of concept

**DOI:** 10.1186/s13567-020-00765-1

**Published:** 2020-03-14

**Authors:** Jorge Garrido-Mantilla, Marie R. Culhane, Montserrat Torremorell

**Affiliations:** Veterinary Population Medicine Department, College of Veterinary Medicine, University of Minnesota, St. Paul, MN USA

## Abstract

The mechanisms of transmission of influenza A virus (IAV) and porcine reproductive and respiratory syndrome virus (PRRSV) in pigs during the pre-weaning period are not fully elucidated. Since viable IAV and PRRSV can be found on the udder skin of lactating sows and the use of nurse sows is a common management practice, we developed a novel nurse sow model to evaluate the transmission of IAV and PRRSV from lactating sows to their adopted piglets. In two studies, we infected pigs with either IAV or PRRSV who then contaminated the udder skin of lactating dams with their nasal and oral secretions while suckling. Once the skin was confirmed virus positive for IAV and PRRSV, the sows were moved to separate empty clean rooms to adopt IAV and PRRSV negative suckling piglets. After adoption, 1 out of eight (12.5%) piglets tested IAV positive 1-day post-adoption (dpa) and the entire litter (8 out of 8) became positive by 4 dpa. In the case of PRRSV, 3 out of 11 (27.3%) pigs tested rRT-PCR positive 2 dpa and there were 7 out of 11 (63.6%) pigs positive at the termination of the study at 7 dpa. This study documented the transmission of IAV and PRRSV between litters of piglets by nurse sows and highlights the importance of the nurse sow-piglet as a unit that contributes to the maintenance of endemic infections in breeding herds. The use of nurse sows in pig farms, though beneficial for minimizing pre-weaning mortality and maximizing farm productivity, is seemingly detrimental as this practice may facilitate the transmission of IAV and PRRSV to piglets prior to weaning.

## Introduction

Influenza A virus (IAV) and porcine reproductive and respiratory syndrome virus (PRRSV) are two of the most important viruses affecting pigs. IAV causes respiratory disease characterized by high morbidity and low mortality with clinical signs of coughing, fever, and sneezing that result in decreased production performance [1]. PRRSV causes reproductive failure leading to abortions, premature farrowings, stillbirths and mummified fetuses, and respiratory disease with interstitial pneumonia in pigs [2]. PRRSV and IAV are commonly found co-circulating in swine herds and together can cause economic losses of up to $10 USD per pig [3].

In recent years, there have been significant efforts to control and eliminate PRRSV from swine breeding herds [4]. Control of IAV has also become a priority as novel viruses of human, avian and swine-origin have become widespread and endemic in U.S. swine herds [5]. The IAV and PRRSV control programs have the common goal of weaning piglets that are virus-free. Piglets prior to weaning are known to be a reservoir for PRRSV and IAV in endemically infected herds [6, 7]. Piglets are born IAV-free but commonly become infected during the suckling period prior to weaning [8]. IAV transmission in pigs occurs mainly by direct contact with virus-laden secretions and exposure to infectious aerosols, although other indirect routes of transmission also exist [8, 9]. In the case of PRRSV, pigs may be born viremic due to in utero infections, or transmission may occur due to contact with infected pigs [10], contaminated materials [11] or aerosols [12]. However, not all sources or the importance of the various transmission routes of IAV or PRRSV infection have been elucidated for piglets prior to weaning, particularly in endemically infected herds and herds undergoing virus control and elimination efforts with enhanced biosecurity practices. Indeed, having PRRSV positive piglets prior to weaning is one of the main challenges for herds attempting to achieve PRRSV-stability, i.e. no detectable PRRSV viremia for 90 days [7]. In addition, there is significant variability in the time it takes for a herd to reach stability and variations in management practices are suspected to contribute to those differences [13]. However, the management practices and on-farm procedures that contribute to or reduce PRRSV infections have not been fully elucidated.

To minimize pre-weaning mortality, it is not uncommon in animals to transfer progeny between dams in order to improve the progeny survivability. The dam that adopts the new progeny is often referred to as a nurse dam. The reasons for transferring the progenies vary and may include the dam’s lack of milking ability, too many animals nursing from a single dam, progenies losing body condition as a result of within litter competition or disease, or farm management protocols that limit the number of animals nursing from a single dam. Similarly, the mixing of animals between litters which is referred to as cross-fostering [14] is also a common practice in the US swine farms for some of the same reasons.

One of the most common practices during the lactation period is to identify a sow with good mothering ability and milk production, wean her biological piglets off of her and have this lactating sow adopt other piglets. We have identified the use of nurse sows as a potential management practice that may facilitate the transmission of IAV and PRRSV between lactating sows and adopted suckling piglets. The use of nurse sows is a standard farm management practice in U.S., in particular in farms with high productivity where approximately 10% of lactating sows will become nurse sows (Allerson, personal communication). Nurse sows are used to adopt piglets at risk of emaciation or mortality. Allowing these piglets to become adopted by and suckle a nurse sow is a way to improve pig livability and maximize pig weight gain through increased milk consumption. The nurse sow may be moved to a room housing the younger pigs or may stay in the same room and younger piglets be brought to her which may depend on the farm management protocols or farm lay-out design. Also, in pigs the practice of cross-fostering (i.e. moving pigs between litters) is common and has been associated with disease transmission between suckling piglets [15].

Our group identified and recently reported the presence of viable IAV and PRRSV on the udder skin of lactating sows [16]. Seventy-eight percent (31 out of 40) of samples collected from the surface of the udder skin of lactating sows from four IAV positive and endemic Midwestern US breeding herds yielded viable IAV [16] indicating that the skin may serve as a source of virus and facilitate IAV transmission between pigs. In humans, IAVs have been detected and isolated from the skin of infected people and direct contact with contaminated hands has been implicated in IAV transmission [17]. Thus, we hypothesize that nurse sows can transmit PRRSV and IAV between litters and effectively perpetuate these infections in piglets prior to weaning. In this study, we evaluated a nurse sow model to test this hypothesis and also evaluated the transmission of these viruses by cross-fostering.

## Materials and methods

### Ethics statement

The protocols in this study were approved by the Institutional Animal Care and Use Committee (Protocol number 1705-34808A) and the Institutional Biosafety Committee (Protocol number 1808-36316H) of the University of Minnesota.

### Experimental design

**Figure 1:**
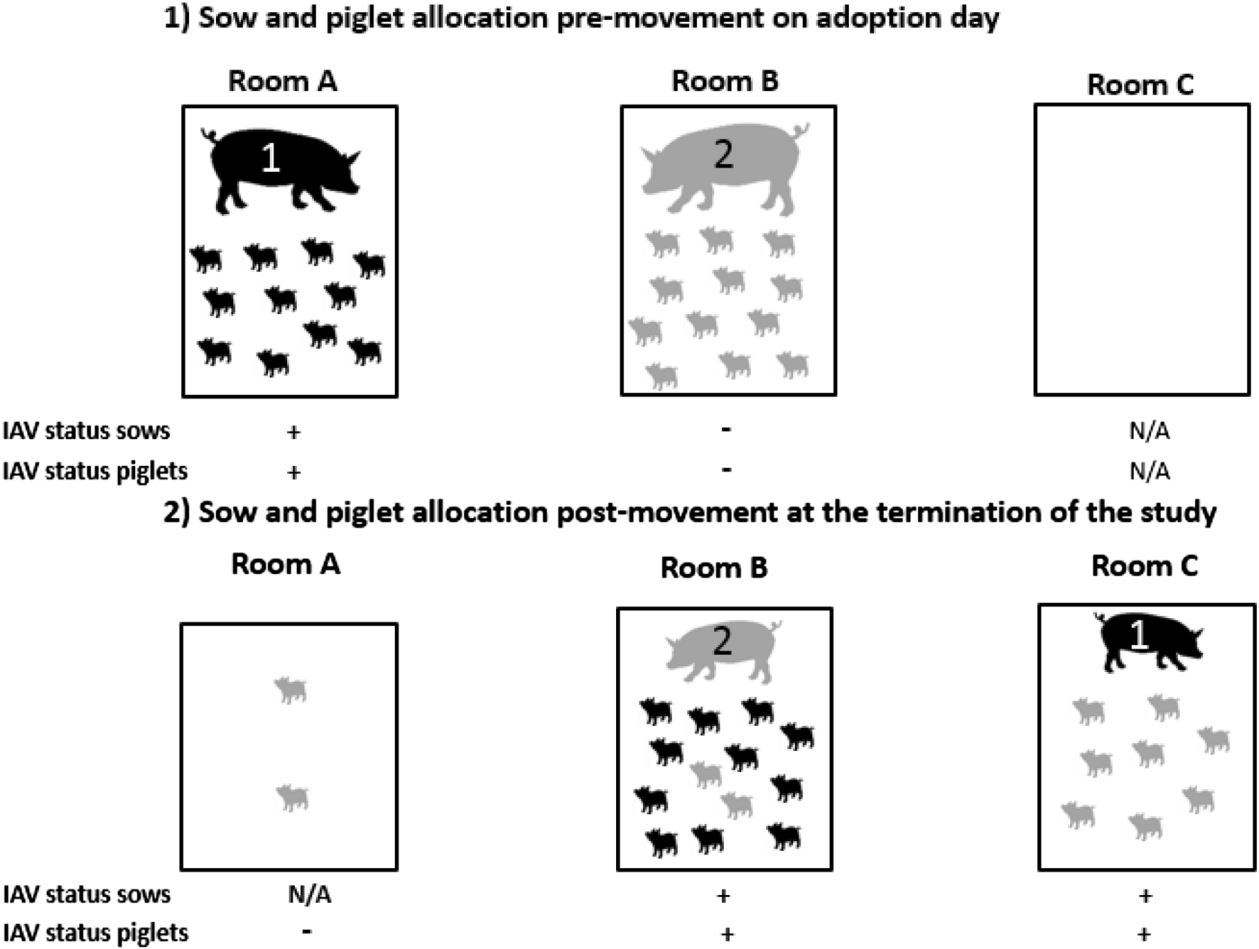
**Influenza A virus (IAV) status of sows and pigs, and sow and piglet room allocation pre-movement on the day when adoption took place (1) and post-movement at the termination of the study (2).** Sow 1 and her eleven piglets are shown in black. Sow 2 and her 12 piglets are shown in grey. Rooms A, B and C indicate the different rooms used during the study. IAV status positive (+) or negative (−) of the sow and litter is shown at the bottom of each room drawing.

A nurse sow model was established by obtaining two IAV- and PRRSV-negative pregnant sows, Sow 1 and Sow 2. Sows tested virus-negative by PRRSV and IAV specific real-time RT-PCR (rRT-PCR) assays and antibody-negative by IAV and PRRSV specific ELISA (enzyme-linked immunosorbent assay) serology tests (ELISA, IDEXX FlockChek™ AI MultiS-Screen Ab Test Kit and ELISA, PRRS X3 Ab Test IDEXX Lab., Westbrook, ME, USA). The sows were housed in two separate BSL-2 rooms at the University of Minnesota–St. Paul campus Animal Research Facilities (Figure 1). Sow 1 farrowed in room A and gave birth to 11 piglets and Sow 2 farrowed in room B and gave birth to 12 piglets. An additional room (C) was used during the study to house the nurse sow (Sow 1) at the time when she adopted her new piglets. This additional room had been cleaned and disinfected and had not had pigs for over 30 days prior to this study and was needed in order to prevent exposure to environmental contamination since the rooms where sows were housed during virus-challenge would have been contaminated. At 6 days post-farrowing, an IAV challenge was performed in order to create a lactating sow with virus-contaminated udder skin yet with limited IAV shedding from the upper respiratory tract, as measured by virus isolation, when adopting the IAV negative pigs. In the case of PRRSV, a challenge model was performed at 19 days post-farrow (or 1 day after the termination of the IAV study) and at time of adoption the nurse sow had contaminated udder skin and suspected viremia. The timing of IAV challenge at 6 days post-farrowing (or 6 days of age for the piglets) was selected conveniently to have suckling pigs old enough to support a viral challenge and still have enough time to have pigs interested in suckling at the time to mimic the adoption process. In the case of PRRSV, 22 days post-farrowing was the earliest after the IAV study had terminated and the pigs would still be interested in suckling. Commonly pigs older than ~ 3–4 weeks of age start showing preference for solid food and they could have stop suckling. If we had waiting much longer the pigs may have not shown the expected behavior associated with the nurse sow/adoption process.

Briefly, for the IAV transmission study, at 6 days post-farrowing (-7 days post-adoption (dpa)) approximately half (*n* = 6) of the piglets from Sow 1 were removed from her and temporarily placed on Sow 2 to suckle. We then intranasally inoculated Sow 1 and her remaining pigs (*n* = 5) with 10^5^ TCID_50_/mL of an H1N1 isolate (A/swine/Iowa/MT_12_07_1920/2012). To confirm IAV infection in Sow 1 and the piglets post-inoculation, we tested nasal swabs and udder skin wipes daily for IAV by rRT-PCR (Table 1). Meanwhile, Sow 2, her 12 biological piglets, and the 6 temporarily placed piglets continued unexposed to IAV in order to preserve their IAV negative status. To ensure the positive presence of IAV on the udder skin of Sow 1, 3 days after the first challenge (−3 dpa) we returned her six temporarily removed pigs to her to be IAV inoculated as described above. When Sow 1 nasal secretions were negative for IAV by virus isolation and IAV rRT-PCR cycle threshold values (ct) were ascending (7 days post-challenge) to levels consistent with minimal nasal shedding of IAV, she was moved to an empty, clean and disinfected room (room C). After relocation and on the same day, Sow 1 adopted eight IAV negative pigs from Sow 2. Similarly, the IAV-negative Sow 2 remained in room B and adopted the eleven IAV positive pigs from Sow 1. This latter movement was done to assess the transmission of IAV from the pigs to the sow. Furthermore, two IAV negative pigs from Sow 2 were left lactating with her and commingled with piglets from Sow 1 in order to evaluate transmission by direct contact, which simulates the farm management practice of cross-fostering. Finally, two IAV negative pigs from Sow 2 were moved into room A which had housed the IAV positive sow and litter and it was now empty in order to evaluate the transmission of IAV by the contaminated environment. Environmental surface wipes were collected at 1, 2.5 and 4 h post-introduction of pigs into room A and pigs were sampled by collecting nasal swabs 1, 2 and 3 days post-placement. Figure 1 shows the distribution of pigs and sows the day before adoption and after movements took place at the termination of the study.

**Table 1:** Influenza A virus reverse transcription polymerase chain reaction (rRT-PCR) and virus isolation (VI) results from sows and litters before and after adoption

Days post-adoption (litter age in days)	Activities	Test	Sow 1			Sow 2		
			Sow nasal swab^a^	Udder wipe	Litter (POS/TOT)	Sow nasal swab	Udder wipe	Litter (POS/TOT)
− 8 (5)	Pre-challenge IAV status confirmation	rRT-PCR VI	NEG NT	NEG NT	0/12 NT	NEG NT	NEG NT	0/13 NT
− 7 (6)	1st IAV challenge of Sow 1 and half of her litter	rRT-PCR VI	NC NC	NC NC	NC NC	NC NC	NC NC	NC NC
− 6		rRT-PCR VI	21 NT	26.95 NT	6/6 NT	NEG NT	NEG NT	0/19 NT
− 5		rRT-PCR VI	22.92 NT	23.73 NT	6/6 NT	NEG NT	NEG NT	0/19 NT
− 4		rRT-PCR VI	30.02 NT	32.49 NT	6/6 NT	NC NC	NC NC	NC NC
− 3 (10)	2nd IAV challenge of Sow 1 and the rest of her litter	rRT-PCR VI	24.59 NT	29.13 NT	NC NC	NC NC	NC NC	NC NC
− 2^b^		rRT-PCR VI	23 POS	24.24 NEG	11/12 NT	NEG NT	NEG NT	0/8 NT
− 1		rRT-PCR VI	28.22 NEG	26.79 POS	12/12 NT	NEG NT	NEG NT	0/8 NT
0 (13)	Adoption^b^	rRT-PCR VI	NC NC	NC NC	NC NC	NC NC	NC NC	NC NC
1		rRT-PCR VI	30.71 NEG	35.21 NEG	1./8^b^ NT	34.83 NEG	23.81 POS	8/12 (2/2)^c^ NT
2		rRT-PCR VI	27.13 NEG	28.01 POS	6./8 NT	26.26 NT	21.18 POS	11/12 (2/2) NT
3		rRT-PCR VI	38.56 NEG	26.04 POS	5./8 NT	30.28 POS	22.16 POS	9/12 (2/2) NT
4		rRT-PCR VI	NC NC	30.69 NEG	8./8 NT	NC NC	NC NC	NC NC
5 (18)	End of IAV study	rRT-PCR VI	NC NC	29.79 NEG	8./8 NT	NC NC	NC NC	NC NC

At time of adoption litters were 13 days of age.

*NC* not collected, *NT* not tested.

^a^Number indicates rRT-PCR cycle threshold value.

^b^Note that on adoption day and thereafter litter results correspond to the newly adopted piglets that originally belonged to the other sow.

^c^In parenthesis, results from in contact pigs after cross-fostering.

**Figure 2:**
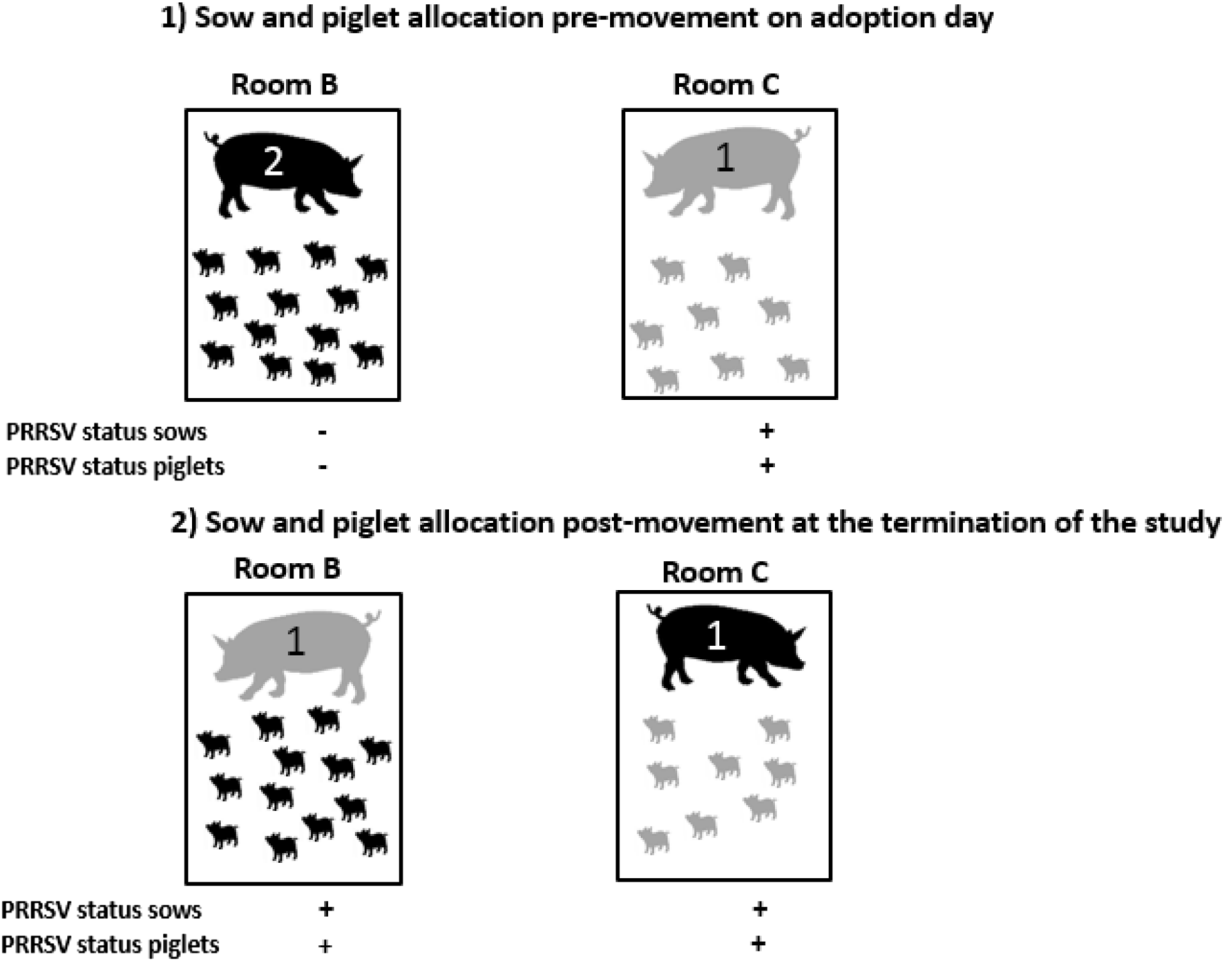
**Porcine reproductive and respiratory syndrome virus (PRRSV) status of sows and pigs, and sow and piglet room allocation pre-movement on the day when adoption took place (1) and post-movement at the termination of the study (2).** Rooms B and C indicate the different rooms used during the study. PRRSV status positive (+) or negative (−) of the sow and litter is shown at the bottom of each room drawing.

For the PRRSV transmission study, after the IAV study was completed Sow 1 piglets now of 22 days of age were intramuscularly inoculated with 3.2 × 10^6^ TCID_50_/mL of PRRSV VR-2332 strain. After confirming that piglets were infected and that the udder skin of Sow 1 tested positive, Sow 1 was moved into room B at 6 days post-challenge to adopt PRRSV negative piglets now of 28 days of age that had remained PRRSV negative with Sow 2 (Figure 2). Prior to movement, the udder wipe collected from Sow 1 tested rRT-PCR positive and had viable virus. Sow 1 was sampled at 1, 2, 4 and 7 days post-adoption by collecting udder skin wipes and serum. The adopted piglets were sampled at days 2, 4 and 7 post-adoption by collecting sera. Figure 2 shows the IAV status and distribution of pigs and sows before adoption on the day of moving the pigs and after adoption at the termination of the study for the PRRSV study.

### Sampling procedures

Nasal swabs were collected for IAV testing from all pigs and sows using rayon‐tipped swab applicators with Stuart’s medium (BBL CultureSwab™ liquid, Stuart single plastic applicator; Becton, Dickinson and Com. Sparks, MD, USA). Collection of nasal swabs was done by inserting the swab approximately 2–4 cm into each nostril and rotating the swab gently. Blood samples were collected for PRRSV testing from sows and pigs by venipuncture of the jugular vein. After collection, serum was separated and kept frozen at − 80 °C. Surface samples from crates, drinkers, and panels were collected with a sterile gauze (described henceforth) and thoroughly wiping the surfaces in contact with the mouth and noses of the pigs. Udder skin wipes were collected by using a 3 × 3 inches sterile gauze impregnated with 10 mL of DMEM-Dulbecco’s Modified Eagle Medium Gibco™ (Grand Island, NY, USA) supplemented with antibiotics and antimycotics and tested for PRRSV and IAV. The gauzes were individually bagged and kept frozen at − 20 °C until use. Udder wipes were collected by wiping the udder skin in the areas of contact with the pigs’ nose and mouth during or after suckling in order to collect the pigs’ oral and nasal secretions. In both studies, all pigs and both sows were sampled at the times shown in Tables 1 and 2.

**Table 2:** Porcine reproductive and respiratory syndrome virus real-time reverse transcriptase polymerase chain reaction (rRT-PCR) and virus isolation (VI) results from sows and litters before and after adoption

Days post-adoption (litter age in days)	Activities	Tests	Sow 1			Sow 2		
			Sow serum^a^	Udder wipe	Litter (POS/TOT)	Sow serum	Udder wipe	Litter (POS/TOT)
− 7 (19)	Pre-challenge PRRSV status confirmation	rRT-PCR VI	NEG NT	NEG NT	NEG NT	NEG NT	NEG NT	NEG NT
− 6 (22)	Challenge	rRT-PCR VI	NEG NT	NEG NT	NEG NT	NEG NT	NEG NT	NEG NT
− 4		rRT-PCR VI	35.81 NEG	27.13 NEG	9/9 NT	NEG NT	NEG NT	NEG NT
− 1		rRT-PCR VI	NC NC	30.4 POS	9/9 NT	NC NC	NEG NT	NC NC
0 (28)	Adoption^b^	rRT-PCR VI	NC NC	NC NC	NC NC	NC NC	NC NC	NC NC
1		rRT-PCR VI	NC NC	35.05 NEG	NC^c^ NC	NC NC	35.15 NEG	NC NC
2		rRT-PCR VI	24.23 POS	33.28 NEG	3/11 NT	22 POS	31.06 NEG	9/9 NT
4		rRT-PCR VI	27.89 NEG	34.99 POS	6/11 NT	22.19 POS	31.84 NEG	9/9 NT
7 (32)	End of PRRSV study	rRT-PCR VI	38.58 NEG	32.58 NEG	7/11 NT	26.32 POS	34.44 POS	9/9 NT

At time of adoption litters were 28 days old.

*NC* not collected, *NT* not tested

^a^Number indicates rRT-PCR cycle threshold value.

^b^Note that on adoption day and thereafter litter results correspond to the newly adopted pigs that were born to the other sow.

### Diagnostic tests

Nasal swabs and udder skin wipes were processed in individual tubes with 2 mL of DMEM-Dulbecco’s Modified Eagle Medium Gibco™ supplemented with antibiotics and antimycotics for viral RNA extraction using the magnetic particle processor procedure (Ambion^®^ MagMAX™AM1835, Viral RNA Isolation Kit; Applied Biosystems, Foster City, CA, USA) and tested by rRT-PCR to detect the IAV matrix gene [18]. For PRRSV, viral RNA was extracted from serum and samples collected from the sow’s udder skin using a commercial RNA isolation kit (QIAamp Viral RNA Mini-Kit, Qiagen, Inc., Valencia, CA, USA) and tested by rRT-PCR to detect the ORF5 gene [19, 20]. IAV and PRRSV rRT-PCR results with cycle threshold (ct) value ≤ 35 were considered positive, ct > 35 and ≤ 40 suspect, and ct > 40 negative.

IAV rRT-PCR positive samples were cultured for virus isolation using Madin–Darby canine kidney (MDCK) cells. MDCK cells were prepared in 6-well plates for each selected sample. Wells were inoculated with 200 µL and 100 µL of the sample, in duplicate, and incubated for 1 h at 37 °C with 5% CO_2_. 1.5 mL of DMEM media (Gibco™) supplemented with 7.5% bovine serum albumin (Gibco™), 1X antibiotic and antimycotic (Gibco™), 750 µL 1 mg/mL trypsin-TPCK, gentamicin, neomycin was added to each well, then these plates were incubated at 37 °C with 5% CO_2_. Plates were evaluated at days 3 and 5 post-incubation for the appearance of positive cytopathic effect (CPE). All the wells with positive CPE were confirmed by hemagglutination assay (HA) using 0.5% turkey red blood cells and VetScan Avian Influenza Type A Virus Rapid Test (Alere Scarborough Inc., Union City, CA, USA). For PRRSV, samples were submitted to the University of Minnesota Veterinary Diagnostic Laboratory and cultured for virus isolation using porcine alveolar macrophages (PAM) and MARC 145 simian kidney cells in 6-well plates for each selected sample. Wells were inoculated with 200 µL of sample and incubated for 5 days. Virus isolation was confirmed by evaluation of cytopathic effect (CPE). A selected number of udder wipes that yielded viable IAV and PRRSV were also titrated to quantify the amount of viable virus on the surface of the udder skin. Titration (TCID_50_/mL) was calculated using the Karber method [21].

## Results

### IAV study

One day prior to adoption, all of the IAV challenged piglets born to Sow 1 were IAV positive in their nasal swabs as was the udder skin wipe of Sow 1, which had a virus titer of 5.6 × 10^6^ TCID_50_/mL. The sow’s nasal swab, however, was virus isolation negative. A summary of the results in chronological order can be seen in Table 1. Conversely, all samples collected from Sow 2 and her piglets prior to the adoption event were consistently IAV rRT-PCR negative.

At one day post-adoption (1 dpa), a single pig was IAV rRT-PCR positive of the eight pigs tested and the entire litter adopted by Sow 1 tested IAV positive by 4 dpa. The virus titer from the udder skin wipe collected at 2 dpa was 1.8 × 10^6^ TCID_50_/mL.

In order to evaluate the effect of cross-fostering and whether IAV positive pigs could serve as a source of infection transmitting IAV to the sow, Sow 2 received all (*n* = 12) the IAV positive pigs from Sow 1 and kept two of her own negative pigs to serve as in contact sentinels. Both Sow 2 and the two sentinel pigs tested IAV rRT-PCR positive 1 day after commingling with the IAV positive pigs (Table 1). Sow 2 tested IAV rRT-PCR positive on the nasal swab (ct 34.83) and udder wipe (ct 23.81), and udder wipes were positive by virus isolation. Interestingly, Sow 2 had lower Ct values in the udder skin than in the nose indicative of higher virus concentration in the udder skin than in the nose.

In room A that had previously housed the experimentally inoculated Sow 1 and her litter, wipes collected from environmental surfaces tested IAV rRT-PCR positive at 1, 2.5 and 4 h post-placement of pigs into the room with rRT-PCR ct values of 30.34, 29.34, and 28.23, respectively. IAV was detected on these surfaces 24, 48, and 72 h later with ct values of 32.57, 34.26, and 35.27, respectively. The pigs that had been placed into room A to evaluate whether the environment could be a source of infectious material tested rRT-PCR negative 72 h after being placed into the room. There was no viable virus isolated from any of the environmental wipes (e.g. the environment tested negative by virus isolation).

### PRRS study

Prior to adoption, Sow 2 piglets tested PRRSV rRT-PCR negative. The serum sample collected from Sow 1 after PRRSV challenge 4 days before adoption tested suspect by rRT-PCR (ct 35.81). The udder skin wipe collected from Sow 1 tested PRRSV positive by rRT-PCR (ct 30.4) and had viable virus with a titer of 5 × 10^3^ TCID_50_/mL at − 1 dpa (Table 2). The udder skin wipe collected from Sow 1 tested PRRSV rRT-PCR suspect (ct 35.05) and positive (ct 33.28) at 1 and 2 dpa, respectively. Udder skin wipes collected from Sow 2 tested positive at 1, 2, 4 and 7 dpa. Three serum samples of the 11 collected from the pigs were PRRSV rRT-PCR positive at 2 dpa. The study was terminated at 7 dpa with 7 out of 11 pigs PRRSV positive. After Sow 2 adopted positive pigs, the PRRSV rRT-PCR test results from udder skin wipes collected from her were suspect (ct value 35.15) at 1 dpa and positive (ct 31.06) at 2 dpa. Sow 2, who tested rRT-PCR negative before the adoption event, herself became rRT-PCR serum positive 2 days after adopting the positive piglets.

## Discussion

Identifying disease transmission pathways is important in order to develop effective disease control and elimination programs. Most production management recommendations to minimize spread of pathogens during lactation focus on suckling pigs rather than sows. In this study, we developed a transmission model to evaluate the role of nurse sows at transmitting IAV and PRRSV between litters. Nurse sows are used to adopt piglets to maximize piglet survivability and herd productivity. Commonly, nurse sows are identified to adopt other pigs at a time when they themselves may be infected or contaminated with pathogens. Furthermore, given the recent results that documented the presence of viable IAV and PRRSV in the udder skin of lactating sows [16], we were interested in evaluating whether nurse sows could have viable PRRSV and IAV in their udder skin and could transmit these two viruses between litters. In this study, we documented the transmission of IAV and PRRSV between litters by nurse sows, revealing the nurse sow-piglet complex as a unit that helps support endemic infections in breeding herds. Our results emphasize the need for management protocols that limit transmission of IAV and PRRSV by nurse sows to suckling piglets during the pre-weaning period.

In order to test our hypothesis, we developed an experimental model to mimic field conditions of nurse sow use. In the case of IAV, we paid special attention to creating a nurse sow that had limited shedding in the upper respiratory tract, as detected by virus isolation, but with udder skin contaminated by oral and nasal secretions shed naturally by the IAV infected suckling piglets. We accomplished this by experimentally infecting the sow and half of her litter in a first challenge followed with a second challenge of the remaining pigs in the litter 4 days later. The second challenge was done to ensure a consistent source of contamination of the sow udder skin at the time when Sow 1 adopted the negative piglets. Our concern was that if we had waited for both Sow 1 and her entire litter to test negative in nasal swabs, there would have been no source of IAV contamination to the udder skin. As shown in the results, at time of adopting the IAV negative pigs, the IAV positive nurse sow tested negative by viral isolation in samples from the upper respiratory tract but had high titers of IAV on the udder skin (5.6 × 10^6^ TCID_50_/mL). Although we cannot fully rule out the possibility that the sow was shedding IAV in her nose given that the Ct value in the nasal swab at time of adoption was 28.22, and that virus isolation is less sensitive than rRT-PCR [22], we believe that a significant source of virus infection to the piglets was the udder skin rather than the secretions from the respiratory tract of the sow. Additionally, we cannot fully rule out the presence of IAV in milk since we did not collect milk samples but detection of IAV in milk is extremely rare [23, 24]. Furthermore, transmission to piglets happened very rapidly with one piglet testing IAV positive 1 dpa and the whole litter by 4 dpa, suggesting that the pigs were exposed to a high quantity of infectious IAV. It is important to consider also that once infection started in a litter, the rapid infection spread within a litter could be the result of pig to pig transmission rather than direct infection from the sow [9]. Transmission of IAV via direct contact with oral and nasal secretions is considered one of the primary routes of transmitting IAV [2]. Thus we showed the importance of the nurse sow/piglet complex in the transmission of IAV prior to weaning.

Similarly, during the PRRSV challenge model, we were also able to mimic the contamination of the udder skin with viable PRRSV by the suckling piglets; however, in this case, we cannot fully rule out the transmission of PRRSV via other means. We suspect the sow may have been viremic at the time of adopting the PRRSV negative piglets given that she was rRT-PCR suspect in blood 4 days prior to adoption and viremic 2 days post-adoption as detected by rRT-PCR and virus isolation, respectively. Furthermore, we cannot rule out the presence of virus in milk, the upper respiratory tract or other bodily secretions given the systemic nature of PRRSV during the acute phase of infection [25]. Nevertheless, the observation that 3 pigs became infected after suckling from a sow that had a moderate titer of 5 × 10^3^ TCID_50_/mL of PRRSV in the contaminated skin is of interest and indicates the possibility of contact transmission via exposure to contaminated skin as part of the nursing process. This process likely occurs independently from transmission taking place in utero during gestation or through other secretions. In our study, only 7 out of 11 pigs tested positive at the termination of the study. Whether there are differences between strains, or age played a role or that we terminated the study too early to see infection of the whole litter, our results agree with other studies were the transmissibility of PRRSV was estimated and obtained a reproduction ratio of 3.53 (CI 2.89–4.18) [26] which shows spread of IAV but not as rapidly as often it is assumed in the field.

PRRSV can cross the placenta and there are significant efforts to have herds stable to PRRSV infection [2]. Stable herds are those that have immune sows to PRRSV (i.e., sows have recovered from infection) and that have piglets born and weaned negative to PRRSV [27]. Our results show that in herds working towards achieving stability, limiting the use of nurse sows may be advantageous. Lastly, we ruled out the environment as being the source of IAV and PRRSV to piglets since adoption of the new litter was done when the nurse sow was moved into a clean room that had been previously cleaned and disinfected and had not contained pigs for a significant period of time.

In this study, we also documented the role of suckling pigs as sources of infection for sows. Although it should not come as a surprise, our study showed that piglets were the source of IAV and PRRSV infection to the negative sows that had adopted them. This is especially relevant for weaned sows, since at weaning these sows are moved into the breeding and gestation barn where they may become in contact with new replacement females (gilts), heat check boars and bred adult sows perpetuating the transmission cycle of viruses from farrowing to breeding/gestation. Further work is needed to evaluate the impact that weaning infected sows has on herd stability. As expected, cross-fostered negative pigs became positive for IAV shortly after coming in contact with positive pigs. Infection of IAV by direct contact is well documented [2] and this study emphasizes the need to minimize piglet cross-fostering practices in order to have a comprehensive IAV control program. We could not document the transmission of IAV from surfaces to the two sentinel pigs, but transmission from the environment to pigs should not be fully ruled out. There is evidence of transmission of influenza via fomites in contaminated environments [28, 29].

The findings of this study show the impact of common management practices such as the use of nurse sows and sow movement in the maintenance and perpetuation of IAV and PRRSV infections. Even though these practices are routine, neither practice is completely identified as a pathway of disease transmission at the farm level, especially not the use of nurse sows, which apparently have production benefits that may outweigh the negative effects of virus transmission. Therefore, this study highlights the importance and contribution of these practices in the continuous circulation of viruses and the presence of diseases; thus, the nurse sow/piglet litter unit should be considered a risk factor for IAV and PRRSV transmission. Nurse sows are selected mostly at weaning at a time when IAV and PRRSV prevalence is high (6) thus it should be reasonable to speculate about the risk of transmitting viruses to younger pigs via nurse sows. Although we identified the nurse sow/piglet unit as an important unit for IAV and PRRSV transmission, further work is needed to understand the relevance of this finding under field conditions, especially in endemic situations where populations may have some level of immunity and transmission may be reduced [30]. Our study was conducted in naïve animals to maximize the likelihood of transmission and given that our sample size was limited and that there were no replicates in the study, we cannot assume, based on these results, what the impact of using nurse sows is under field conditions.

In conclusion, this study identified nurse sows as a source of IAV and PRRSV transmission during the suckling period and highlighted the role that the nurse sow/piglet unit has in maintaining endemic infections in sow farms. Furthermore, this study highlights how a management practice can contribute to the dissemination and perpetuation of IAV and PRRSV infections and emphasizes the need to evaluate intervention strategies directed at minimizing transmission of pathogens via nurse sows. This study also supports prior findings that viable viruses can be found in the udder skin of lactating sows. Overall, reducing infection of sows during lactation and piglets while suckling is necessary to minimize the spread of diseases to other farms when pigs are moved at weaning.

## Data Availability

The datasets used and/or analysed during the current study are available from the corresponding author on a reasonable request.

## Abbreviations

IAV: Influenza A virus
PRRSV: Porcine reproductive and respiratory syndrome virus
Dpa: Day post-adoption
DAP: Deposition of airborne particle
rRT-PCR: Reverse transcription polymerase chain reaction
MDCK: Madin–Darby canine kidney
RNA: Ribonucleic acid
BSL-2: Biosecurity level two
TCID_50_: Median tissue culture infectious dose
